# Omar Tawfik, MB Ch B, MsC, M.D

**Published:** 2009

**Authors:** Salah N. El-Tallawy

**Affiliations:** *Minia University, Egypt*

1942-2009

I knew Professor Tawfik since 1989. For all the years that I have known him, he was a wonderful person and an eminent professor. He was a great friend and a kind human being, with a great vision and hard-working attitude. He was a leader in pain medicine in Africa and the Middle East. He has trained hundreds of physicians from Egypt and Middle East in pain medicine. Professor Tawfik was one of the original leaders of pain medicine all over the world. He was the President of the Egyptian Society of Pain, Middle East Society of Pain, Founder of the Pan Arab Federation of Pain, and Editor-in-chief of the Pain Journal. He was a Council Member of the ‘IASP’, ‘WIP’, ‘WSPC’, and Advisor to the WHO. Throughout our life we come across many people, but only one who shares our problems and opinion. Professor Tawfik was my leader and always will be. I pray God to give his wife and daughters the strength to bear the loss.

